# Chloride in intensive care units: a key electrolyte

**DOI:** 10.12688/f1000research.11401.1

**Published:** 2017-11-01

**Authors:** Ghassan Bandak, Kianoush B. Kashani

**Affiliations:** 1 Multidisciplinary Epidemiology and Translational Research in Intensive Care (METRIC) Research Group, Mayo Clinic, Rochester, MN, USA; 2 Division of Pulmonary and Critical Care Medicine, Department of Internal Medicine, Mayo Clinic, Rochester, MN, USA; 3 Division of Nephrology and Hypertension, Department of Internal Medicine, Mayo Clinic, Rochester, MN, USA

**Keywords:** acute kidney injury, chloride abnormalities

## Abstract

Over the past few years, chloride has joined the league of essential electrolytes for critically ill patients. Dyschloremia can occur secondary to various etiologic factors before and during patient admission in the intensive care unit. Some cases are disease-related; others, treatment-related. Chloride abnormalities were shown in animal models to have adverse effects on arterial blood pressure, renal blood flow, and inflammatory markers, which have led to several clinical investigations. Hyperchloremia was studied in several settings and correlated to different outcomes, including death and acute kidney injury. Baseline hypochloremia, to a much lesser extent, has been studied and associated with similar outcomes. The chloride content of resuscitation fluids was also a subject of clinical research. In this review, we describe the effect of dyschloremia on outcomes in critically ill patients. We review the major studies assessing the chloride content of resuscitation fluids in the critically ill patient.

## Introduction

Chloride constitutes 70% of the negative ion content of the body ^1, 2^. The principal dietary chloride intake is in the form of salt and thus nutritional deficiencies of chloride are rare ^3^. Chloride and sodium have a significant role in maintaining osmolarity, acid-base balance, and electro-neutrality of body fluids ^1, 4^. The various mechanisms and hormones that regulate sodium and volume balance also regulate chloride concentration, including the renin-angiotensin-aldosterone system, sympathetic nervous system, atrial natriuretic peptide, and other factors affecting renal blood flow ^3^. The chloride ion distributes in all body fluid compartments and mainly in the extracellular fluid compartment of plasma and interstitial fluid. Its concentration in the intracellular fluid compartment is less than in extracellular fluid ^2^.

Chloride is frequently reported among other daily laboratory values obtained for critically ill patients. However, it attracts only minimal attention beyond its role in acid-base calculations. Interestingly, some reports indicate that dyschloremia may be associated with harmful effects. In animal models, hyperchloremic acidosis has been shown to have deleterious effects, such as an increase in inflammatory markers in animal models of sepsis and a reduction in mean arterial pressure in septic rats when pH and chloride levels have changed ^5, 6^. In other animal experiments, high chloride concentration was associated with increased renal vasoconstriction and reductions in renal blood flow and glomerular filtration rate ^7, 8^.

In recent years, these findings have led to growing interest in the interplay of chloride and its effect for the critically ill patient. Both hypochloremia and hyperchloremia have been associated with worse clinical outcomes, including death and acute kidney injury (AKI). The chloride concentration in resuscitation fluids in the intensive care unit (ICU) was determined to be a clinically relevant field of research. In this review, we outline the role of chloride in the critically ill patient and discuss the recent literature behind that role.

## Dyschloremia in the ICU

Hypochloremia in the ICU can be due to pathophysiologic processes related to the illness or secondary to therapeutic interventions ^1, 9^. The leading causes of hypochloremia are related to gastrointestinal or renal losses of chloride ions ( Table 1). Renal losses of the chloride ion can occur in the clinical setting of diuretic use ^9^ or, less commonly, in renal disorders such as Bartter syndrome. Gastrointestinal losses can occur through losses of chloride-rich fluids (for example, vomiting). Hypochloremia can also develop with excess water gains (for example, syndrome of inappropriate antidiuretic hormone secretion and congestive heart failure) ^1, 2^. The reported prevalence of hypochloremia has varied according to the clinical setting and patient population. In the general ICU setting, different studies have reported an incidence between 6.7% and 37% ^10– 13^. Among patients with heart failure, the reported incidence varied from 13% to 23% ^14– 16^.

**Table 1.** Causes of hypochloremia.

Mechanism	Loss location	Example
Chloride loss	Gastrointestinal	Vomiting Gastric fluid drainage High-volume ileostomy drainage
	Renal	Diuretic use Bartter syndrome Gitelman syndrome
Excess water gain (compared with chloride)	Congestive heart failure Syndrome of inappropriate antidiuretic hormone	Infusion of hypotonic solutions
Excess sodium gain (compared with chloride)		Infusion of sodium bicarbonate

Hyperchloremia, in contrast to hypochloremia, has been in the medical spotlight recently. The rates of occurrence of hyperchloremia in the ICU vary widely according to study population and time of measurement. Mechanisms leading to the development of hyperchloremia include the iatrogenic mechanism from excessive chloride administration during resuscitation with chloride-rich solutions. Excessive water loss, either net water loss or in excess of chloride loss, in addition to increased renal reabsorption of chloride, is another causative mechanism ( Table 2).

**Table 2.** Causes of hyperchloremia.

Mechanism	Loss location	Example
Chloride administration		Chloride-rich intravenous fluids
		Total parenteral nutrition
Water loss (true water loss or relative to chloride)	Renal	Diabetes insipidus
		Diuretic use
		Osmotic diuresis
		Postobstructive diuresis
	Extrarenal	Fever
		Hypermetabolic state
		Diarrhea
		Burns
		Exercise and severe dehydration
Definitive or relative increase in tubular chloride reabsorption		Renal tubular acidosis
		Renal failure
		Acetazolamide use
		Ureteral diversion procedure
		Post-hypocapnia

## Associations of dyschloremia and outcomes in the ICU

Hypochloremia has gained minimal attention in the literature, and its related data are scarce. Tani and colleagues ^12^ reported the association of hypochloremia with increased ICU length of stay and death in a mixed surgical and medical ICU setting. The authors, following multivariate analysis, stated that hypochloremia was not an independent predictor of mortality. Nonetheless, they showed a significant correlation between hypochloremia and higher Acute Physiology and Chronic Health Evaluation scores. In another report, Kimura and colleagues ^11^ found that hypochloremia within the first 48 hours postoperatively was independently associated with an increased mortality rate compared with patients who had normal chloride levels, even after adjustment for illness severity. Shao and colleagues ^10^ looked into the relationship of dyschloremia—hypochloremia and hyperchloremia—and AKI, as well as other outcomes, in a large cohort of ICU patients. In this cohort, the incidence of hypochloremia before ICU admission was high and reported as 37%. They found that baseline hypochloremia (serum chloride of less than 94 mmol/L) was an independent risk factor for the development of AKI compared with normochloremia. They also noted longer ICU and hospital lengths of stay as well as increased mortality rate in the presence of hypochloremia. In another study, authors demonstrated an association between hypochloremia and longer use of non-invasive ventilation in patients with exacerbation of chronic obstructive pulmonary disease ^17^. Not only hypochloremia but the rate of increase in chloride level has been found to be associated with worse outcomes among hospitalized patients ^18^.

Many recent studies highlighted the potential effects of hyperchloremia on patient outcomes. The association between hyperchloremia and death in adult critically ill patients was first reported in 2011; Boniatti and colleagues ^19^ noted that, in a prospective cohort of 175 patients, the addition of serum chloride to multiple logistic regression models improved the accuracy of the models to assess the risk of death. Various other reports have further highlighted the association between hyperchloremia with worse outcomes. Neyra and colleagues ^20^ found that, among hyperchloremic patients with severe sepsis or septic shock, a higher chloride level at 72 hours after ICU admission was associated with a higher mortality rate. They also noted an increase in death (odds ratio of 1.37) with each 5 mEq/L increase in chloride level among those patients. Interestingly, they did not find a relationship between ICU admission chloride level and death. Likewise, in trauma patients, hyperchloremia 48 hours after ICU admission and a change in chloride levels were independent predictive factors of the 30-day mortality rate ^21^. Other reports found similar associations with minimal differences. After non-cardiac surgery, for patients with normal preoperative serum chloride level, death was associated with postoperative hyperchloremia ^22^, and in a mixed ICU population, higher mortality rates were associated with ICU admission hyperchloremia though not with patients undergoing elective cardiac surgery ^13^.

Of the earlier reports on AKI and hyperchloremia, Zhang and colleagues ^23^ found an association between maximum chloride level and AKI incidence. However, chloride level on ICU admission was not associated with AKI development. They also found that mean chloride levels were higher among patients who had AKI. Shao and colleagues ^10^ also found an association of hyperchloremia and AKI. Another study found an increase in AKI rate among septic patients with a moderate increase in serum chloride (a change of at least 5 mmol/L) even without hyperchloremia ^24^. However, not all studies revealed such a direct relationship. A study of patients with ST-elevation myocardial infarction undergoing percutaneous interventions failed to demonstrate a similar association between hyperchloremia and AKI ^25^.

## Use of chloride-rich fluids in the ICU

The choice of the optimal crystalloid fluid in the ICU has been debated lately. The interplay of chloride imbalance and critical illness has been a driver of such conversation. Randomized controlled trials have addressed the conundrum of using chloride-rich solutions (0.9% saline) or more chloride restriction and balanced crystalloid solutions (for example, Ringer’s lactate and PlasmaLyte) ^26– 28^. Several patient populations have been studied, and various outcomes, such as death, AKI, nutrition, and even coagulopathy and transfusion requirements, have been addressed. Several published randomized clinical trials relayed the effect of chloride concentration in resuscitation fluids and the outcomes of critically ill patients. Unfortunately, most of the older published trials had small sample sizes (<100 patients) ^26, 27^ or included patients with less severity of illness.

Yunos and colleagues ^29^ performed a prospective, open-label, sequential period trial showing that the use of chloride-restrictive intravascular fluid strategy for resuscitation, versus a chloride-rich strategy, reduced AKI incidence and the need for renal replacement therapy (RRT) among ICU patients. However, the authors did not find a positive correlation with ICU or hospital death or lengths of stays. They also did not report chloride levels in their patients; therefore, a clear association between baseline dyschloremia and a change in chloride level could not be established. In another study ^30^, 150 patients who underwent cadaveric renal transplant were randomly assigned to receive normal saline or an acetate-buffered, balanced crystalloid during and after transplant surgery. The research arm receiving the balanced crystalloid had less hyperchloremia and acidosis and required fewer catecholamines. These trials raised concerns about the potential harmful associations with the use of chloride-rich solutions.

Within the past two years, three very relevant trials which provided valuable perspectives on this topic were published. In 2015, Young and colleagues ^31^ published the results of the SPLIT (0.9% Saline vs. PlasmaLyte 148 for ICU Fluid Therapy) trial, a randomized double-blinded controlled trial on the use of saline versus a buffered crystalloid (PlasmaLyte 148; Baxter Healthcare Ltd). They enrolled 2,278 ICU patients, of whom 2,092 were included in the primary analysis. These investigators did not find a significant difference between treatment arms in the development of AKI, which was their primary outcome. They also did not find a significant difference in hospital death or RRT. Although this is probably the largest randomized trial on this topic to date, the authors did not report chloride levels. In addition, the average positive fluid balance was slightly less than 2 L, and most of the enrollees were surgical patients with fewer comorbidities. Because an adverse effect of a high chloride level on kidney function is mainly reported in sicker patients with higher serum chloride concentrations, an explanation for this study’s negative findings is the demographic characteristics of the enrolled patients and the limited exposure to the potentially nephrotoxic intravascular solution (with high chloride concentration) in the intervention arm.

The isotonic Solution Administration Logistical Testing (SALT) trial was published in 2017 ^32^. This was a pilot trial for a much larger, pragmatic, cluster-randomized, multiple-crossover study aiming to enroll 14,000 patients. The study involves alternating crystalloid assignment to different ICUs on a monthly basis following a cluster-randomized crossover design. The primary outcome of the larger project will be major adverse kidney events by hospital discharge or day 30 (MAKE 30), including in-hospital mortality, receipt of new RRT, or persistent renal dysfunction defined as a final inpatient serum creatinine value of at least 200% of the baseline serum creatinine concentration. The pilot enrolled close to a thousand patients and revealed that the selected design could produce well-balanced groups with separation in the type of crystalloid received. Their patient-centered outcomes showed no difference between the groups in the rate of MAKE 30. Comparing the patient populations of the SALT and SPLIT trials is noteworthy as the patients in the SALT trial were admitted to the ICU mainly for sepsis or respiratory failure as opposed to mostly surgical patients in the SPLIT trial. Nonetheless, both studies had low fluid administration volumes. In the SALT and SPLIT trials, the median volumes of crystalloid received in both arms were less than 1,500 and 2,000 mL, respectively. Interestingly, in the subgroup analysis of patients who received larger volumes, the SALT investigators did find a significant difference in the rate of MAKE 30 between treatment arms.

The Limiting Intravenous Chloride to Reduce AKI (LICRA) trial is among the latest evidence on this topic ^33^. The investigators studied the effect of limiting chloride load in patients who underwent cardiac surgery. They designed a prospective multiple sequential period study which included the use of chloride-rich fluids during the first and last periods. Normal saline and 4% albumin were considered to be fluids with high chloride content (used in the first and fourth periods). They included Hartmann’s solution and 20% albumin in the second period and PlasmaLyte and 20% albumin in the third period. Fluid administration was controlled during the perioperative and early postoperative periods. The average volume administration was close to 5 L of crystalloid and colloid. However, the average chloride load was higher in the chloride-rich group. They found no difference among the groups in their co-primary outcomes of change in serum creatinine on postoperative day 5 and AKI stage 2 or 3 development according to the Kidney Disease: Improving Global Outcomes (KDIGO) creatinine AKI definition criteria ^34^. Likewise, the groups did not differ in the incidence of postoperative RRT, hospital mortality, or ICU length of stay. Hyperchloremia and acidemia occurred more frequently in the chloride-rich group, while the chloride-limited group developed more alkalemia, hypokalemia, and hyponatremia. Interestingly, more than 60% of patients in the chloride-restricted strategy developed hyperchloremia.

## Conclusions

In a review of the currently available literature on dyschloremia in the ICU, one should remember that most studies are retrospective or small. In some of the larger studies on the use of normal saline, chloride levels were not reported ^29, 31^. The studies looking into associations of hyperchloremia and outcomes were very heterogeneous with different measured associations and various outcomes. The association between hyperchloremia and death was not always observed or reported. Furthermore, associated electrolyte imbalances from fluid resuscitation—whether the perceived worse outcomes such as death or AKI are related to hyperchloremia per se or to rapid chloride loading with its associated acid-base imbalance—have yet to be carefully examined. Dyschloremia reflects a pathologic process or is a result of therapeutic interventions. Close monitoring of chloride concentration among critically ill patients who undergo multiple interventions may be considered as an important step. In addition, isolation of the dyschloremia effect in the absence of the driving cause may be challenging and less informative.

Following the examination of the body of literature about the consequences of chloride-rich versus more balanced solutions on critically ill patients, we noted that earlier studies suggested that the use of balanced fluids may be associated with less AKI and need for RRT. However, the more recent trials failed to show a significant effect on death or AKI. The use of low volumes of intravenous fluids administered in treatment arms of the SALT and SPLIT trials among patients with less comorbid conditions may explain the failure to observe such relationships. In contrast to these trials, the LICRA trial involved the use of a larger volume of fluid administration yet did not reveal any significant difference in study outcomes, keeping in mind hyperchloremia occurred in more than half of the patients in the chloride-restricted arm.

What seems to be an acceptable general practice among intensivists is to avoid chloride-rich solutions in non-hypochloremic patients. Although patients with hypovolemic metabolic alkalosis or intracranial hypertension may benefit from higher chloride concentration during fluid resuscitation, those who have hyperchloremia, increased risk of AKI, or metabolic acidosis may be harmed by chloride-rich solutions. Clinicians intuitively favor the use of physiologic solutions when resuscitating a critically ill patient. However, to date, no robust data indicate a clear benefit from this strategy. We suggest that, during intravenous fluid resuscitation, clinicians treat different solutions as other medications and consider the benefits and potential adverse effects before prescribing any solution.

## Future research

Prospective trials with more precise design may clarify the answer related to the impact of chloride changes on patient outcomes. These can include the use of chloride level to guide fluid therapy, prospective investigations to assess the impact of dyschloremia at baseline or resultant from therapeutic interventions, study of the potential role of chloride-rich fluids in hypochloremic or normochloremic patients during early resuscitation, and studying the effects of different fluid management strategies in each specific group of patients rather than heterogeneous populations in the hospital or ICUs. Such research would result in more relevant conclusions.
